# Nonylphenol Polyethoxylates Enhance Adipose Deposition in Developmentally Exposed Zebrafish

**DOI:** 10.3390/toxics10020099

**Published:** 2022-02-20

**Authors:** Christopher D. Kassotis, Matthew K. LeFauve, Yu-Ting Tiffany Chiang, Megan M. Knuth, Stacy Schkoda, Seth W. Kullman

**Affiliations:** 1 Institute of Environmental Health Sciences and Department of Pharmacology, Wayne State University, Detroit, MI 48202, USA; mlefauve@wayne.edu (M.K.L.); yutingtc@wayne.edu (Y.-T.T.C.); 2 Lineberger Comprehensive Cancer Center, University of North Carolina School of Medicine at Chapel Hill, Chapel Hill, NC 27514, USA; mmknuth@ad.unc.edu; 3 Department of Genetics, University of North Carolina School of Medicine at Chapel Hill, Chapel Hill, NC 27514, USA; 4 Toxicology Program, North Carolina State University, Raleigh, NC 27695, USA; sschkod@ncsu.edu (S.S.); swkullma@ncsu.edu (S.W.K.)

**Keywords:** endocrine disrupting chemicals, adipogenesis, alkylphenol ethoxylates, ethoxylated surfactants, mixtures, obesogen

## Abstract

Alkylphenol polyethoxylates (APEOs), such as nonylphenol ethoxylates (NPEOs), are high-production-volume surfactants used in laundry detergents, hard-surface cleaners, pesticide formulations, textile production, oils, paints, and other products. NPEOs comprise −80% of the total production of APEOs and are widely reported across diverse environmental matrices. Despite a growing push for replacement products, APEOs continue to be released into the environment through wastewater at significant levels. Research into related nonionic surfactants from varying sources has reported metabolic health impacts, and we have previously demonstrated that diverse APEOs and alcohol polyethoxylates promote adipogenesis in the murine 3T3-L1 pre-adipocyte model. These effects appeared to be independent of the base alkylphenol and related to the ethoxylate chain length, though limited research has evaluated NPEO exposures in animal models. The goals of this study were to assess the potential of NPEOs to promote adiposity (Nile red fluorescence quantification) and alter growth and/or development (toxicity, length, weight, and energy expenditure) of developmentally exposed zebrafish (*Danio rerio*). We also sought to expand our understanding of the ability to promote adiposity through evaluation in human mesenchymal stem cells. Herein, we demonstrated consistent adipogenic effects in two separate human bone-marrow-derived mesenchymal stem cell models, and that nonylphenol and its ethoxylates promoted weight gain and increased adipose deposition in developmentally exposed zebrafish. Notably, across both cell and zebrafish models we report increasing adipogenic/obesogenic activity with increasing ethoxylate chain lengths up to maximums around NPEO-6 and then decreasing activity with the longest ethoxylate chain lengths. This research suggests metabolic health concerns for these common obesogens, suggesting further need to assess molecular mechanisms and better characterize environmental concentrations for human health risk assessments.

## 1. Introduction

Alkylphenol polyethoxylates (APEOs), such as nonylphenol ethoxylates (NPEOs), are high-production-volume nonionic surfactants used in laundry detergents, hard-surface cleaners, pesticide formulations, textile production, oils, paints, and other products [1,2,3,4]. NPEOs comprise −80% of the total production of APEOs, with octylphenol ethoxylates (OPEOs) comprising most of the rest [5]. High efficiency and low costs promoted global annual nonionic surfactant production of >13 million metric tons in 2008 [6] and >USD 33 billion in global revenues in 2014 [7]. NPEOs are formed through reactions of base alkylphenols with ethylene oxide, resulting in varying degrees of ethoxylation. Ethoxylate chains shorten over time in the environment and during water treatment [8,9,10], and APEOs eventually degrade into the alkylphenol hydrophobes; these base alkylphenols, such as nonylphenol, have well-described toxicity [11,12,13]. As a result of this described toxicity, there has been a growing movement to substitute APEOs with alcohol ethoxylates (AEOs), which are generally considered benign surfactants, with minimal toxicity for parent compounds and metabolites [14]. Despite this increasing use of replacements, NPEOs are still widely reported in environmental samples, including municipal wastewater [15,16], finished drinking water [17], sediment samples [4,18], in unconventional oil and gas wastewater [19,20,21], and with high frequencies in residential indoor household dust samples [22]. These studies indicate that APEOs continue to be released into the environment at ng/L–μg/L levels and accumulate in various biota [4,23,24,25,26,27], resulting in various adverse health impacts (e.g., growth and development, reproduction, gross lethality, etc.) [28,29,30,31,32], though insufficient internal measurements APEOs exists in large part due to analytical challenges associated with their detection.

Limited research has suggested in vivo metabolic health impacts for APEOs. Researchers assessing alkylphenols and short-chain APEOs reported persistent increased body weight and/or growth rate at 160–466 days following exposure to environmentally relevant concentrations (1–30 μg/L) in juvenile rainbow trout [33], particularly for the nonylphenol mono-carboxylic acid. Others have reported endocrine impacts on reproduction, development, and fertilization at concentrations ≥10 μg/L in diverse fish species [14,28,29,30,31,32]. Research on similar compounds demonstrated that Tween 80 and Span 80, nonionic surfactants, and dioctyl sodium sulfosuccinate (DOSS), an anionic surfactant (components of Corexit 9500 and structurally similar to the NPEOs examined herein), were able to act as adipogens in vitro and/or obesogens in vivo (rodents) [34,35], though presumably through distinct mechanisms. To further assess potential adipogenic effects of ethoxylated surfactants, we previously demonstrated adipogenic effects of APEOs and AEOs using the 3T3-L1 cell line [36]. We demonstrated that both APEOs (including both NPEOs and OPEOs) and AEOs promoted significant adipogenic activity, with NPEO and cetyl alcohol ethoxylate inducing the greatest magnitude of effects (>200% triglyceride accumulation relative to the maximal rosiglitazone control induced response, 100–150% increased DNA content relative to the differentiated solvent control). Interestingly, these effects appeared to be specific to the ethoxylate chain length, with the base hydrophobes inducing limited or no adipogenic activity themselves [36]. When evaluating the length of the NPEO ethoxylate chain lengths, we reported increasing adipogenic activity with increasing ethoxylate chain length up to an average length of 6, and then decreasing activity with further increases in chain length up to an average of 20 ethoxylate chains. While these results suggest robust adipogenic effects, we also recently published research demonstrating poor inter-laboratory reproducibility in chemical responses for the 3T3-L1 cell model [37], suggesting a need to substantiate this testing in more human relevant in vitro models. Moreover, we still have limited understanding of these effects in whole organisms, particularly for varying ethoxylate chain lengths.

Zebrafish have emerged as a crucial model for metabolic health research [38]. They have quick development, transparent bodies, and morphologically similar adipose to humans. Zebrafish store neutral triglycerides in lipid droplets within white adipocytes similarly to mammals, and exhibit similar gene expression associated with adipocyte differentiation, lipolysis, and endocrine function [39]. Given these characteristics, zebrafish are readily amenable to fluorescent staining and full body fluorescent imaging to characterize and quantify the 34 anatomically, physiologically, and molecularly distinct depots throughout their bodies [40,41,42]. Adipocytes can begin to develop as early as 8 days post-fertilization (dpf), beginning in the pancreatic and abdominal visceral adipose depots (more established adipose depot in this region by −15 dpf), followed by a variety of cranial and ocular subcutaneous adipose depots, and subsequently developing throughout the rest of the fish [40,41,42]. Ten years ago, Tingaud-Sequeira et al. developed a clear protocol for evaluating putative obesogenic chemicals using zebrafish [39]; this test utilizes larval exposures and can evaluate both diet-induced and chemical exposure-induced adiposity in short time frames [43,44,45]. Since then, diverse publications have utilized zebrafish and a range of exposure protocols to demonstrate altered growth trajectories and adipose development following exposure to diverse control chemicals and environmental contaminants [46,47,48,49,50,51].

There is a need for more tractable models of assessing obesity such as zebrafish, particularly given the call for reduced mammalian vertebrate animal model use. Numerous environmental contaminants have been demonstrated to directly modulate metabolic health endpoints, via adipogenic outcomes (triglyceride accumulation and/or pre-adipocyte proliferation) in vitro and/or altered growth trajectories, weight, and adiposity in animal models [52,53]. Metabolic disorders, such as obesity, have been increasing in incidence over the last several decades, concurrent with a rapid rise in industrial chemical production. In the US, 8.9% of infants and toddlers [54,55], 18.5% of 2–19 year old’s [54,55], and 42.4% of adults (20+) [56] are classified as obese, with even larger proportions classified as overweight. High societal and health care costs [57,58], along with poor performance of potential interventions, have driven support for research into other putative causal factors. There is now a growing body of research demonstrating a causal role for metabolism-disrupting chemicals (MDCs), which can modulate metabolism in vivo and/or triglyceride accumulation in vitro [52,53]. Specifically, NPEOs have received limited attention on metabolic health outcomes, particularly for the longer ethoxylate chain compounds. Despite this, they are present nearly ubiquitously throughout the environment [4,15,16,17,18,19,20,21,22], as noted above.

The goals of this study were to assess the potential of NPEOs to promote adiposity and alter growth and development of exposed zebrafish. Specifically, while metabolic disruption has been demonstrated for the base nonylphenol and certain short chain length ethoxylates, very limited data is available on longer chain length polyethoxylates, and how the varying ethoxylate chain lengths may influence the obesogenic effects for these compounds. We sought to expand our understanding of the ability to promote adiposity through evaluation in two separate human mesenchymal stem cell (hMSC) models. We previously assessed a range of NPEOs from the base hydrophobe up to an average ethoxylate chain length of 20 using the murine 3T3-L1 pre-adipocyte model to rigorously assess these contaminants for potential impacts on metabolic health outcomes [36]. Based on this testing, we hypothesized that a medium ethoxylate chain length (6–10 ethoxymers) might promote the greatest adipogenic/obesogenic effects in these models.

## 2. Materials and Methods

### 2.1. Chemicals

Chemicals used are described in detail in Table 1, including CAS numbers (where available), sourcing information, and basic physicochemical properties. Stock solutions were prepared in 100% DMSO (Sigma cat #D2650), using either the molecular weight or average molecular weight (ethoxylated surfactants), and stored at −20 °C between uses.

### 2.2. Cell Care 

Human bone marrow mesenchymal stem cells (hMSCs) were purchased from two vendors: Zenbio (catalog #HBMMSC-F; lot #HBMMSC071819A; passage 3) and Lonza (catalog #PT-2501; lot #155677; passage 2). Cells were maintained as suggested by manufacturer in manufacturer-specific mesenchymal stem cell basal growth media for Zenbio (catalog #BMSC-1) and for Lonza (catalog #PT-3001). Cells were maintained in a sub-confluent state until differentiation and each thaw of frozen cells was utilized within three passages, with no appreciable modulation in response observed.

### 2.3. Adipogenic Differentiation and Outcome Measurements in hMSCs

Zenbio and Lonza hMSCs were induced to differentiate according to manufacturer’s instructions. Briefly, cells were seeded in provider-specific basal media into 96-well tissue culture plates (Greiner catalog #655090) at approximately 30,000 cells per well. Once confluent, differentiation was induced using the cell line providers’ commercially available differentiation media. Media was replaced with test chemicals and/or controls using a 0.1% DMSO vehicle in differentiation media (Zenbio catalog #DM-2-500; Lonza catalog #PT-3102B). For Zenbio-sourced cells, plates were left undisturbed for seven days and then media was removed and replaced with fresh dilutions of test chemicals and/or controls using a 0.1% DMSO vehicle in adipocyte maintenance media (Zenbio catalog #AM-1) for a further seven days and were left undisturbed until day fourteen (Figure S1A). Lonza cells were differentiated with three rounds of differentiation induction, according to company instructions. Specifically, cells were treated with differentiation media and test chemicals for three days, then switched to adipocyte maintenance media (Lonza catalog #PT-3102A) for three days. This cycle was repeated twice more (three days differentiation media and three days adipocyte maintenance media, each with fresh test chemical dilutions) and then maintained in adipocyte maintenance media, with media and test chemical changes every 3–4 days, until 21 days after the initial induction, when plates were assayed (Figure S1B). Differences in total time of differentiation and varying differentiation windows reflect differences in recommended protocols by the two cell line providers (Lonza and Zenbio).

Plates were assayed for triglyceride accumulation and DNA content after fourteen days (Zenbio cells) or twenty-one days (Lonza cells) of differentiation as described previously [59,60]. Media was removed and cells were rinsed with Dulbecco’s phosphate-buffered saline (DPBS; Gibco cat #14040). Each well was then replaced with 200 μL of dye mixture per plate, (20 mL dye mixture preparation: 19 mL DPBS, 20 drops NucBlue^®^ Live ReadyProbes^®^ Reagent (Thermo cat #R37605), and 500 μL Nile Red solution (40 μg/mL solution; Sigma cat #72485-100MG)). Plates were incubated, protected from light, for approximately forty minutes at room temperature, then fluorescence was read using a Molecular Devices SpectraMax iD5 microplate spectrofluorimeter: excitation 485 nm/emission 572 nm for Nile Red and 360/460 for NucBlue. Triglyceride accumulation was calculated as percent activities relative to the maximal rosiglitazone-induced fold induction over the intra-assay differentiated vehicle control responses (0.1% DMSO). Given the large disparities among adipogenic differentiation protocols, the extent of solvent control differentiation can be widely different across laboratories; as such, comparison to rosiglitazone-induced maximal responses provide a more robust and comparable measure for evaluating consistency of effects across laboratories. DNA content was calculated as percent deviation from the differentiated vehicle control responses and was then used to normalize total triglycerides per well to obtain triglycerides per unit DNA (used as proxy measure for triglycerides per cell). DNA content measurements can reflect either pre-adipocyte proliferation (an adipogenic response; positive values) or cytotoxicity (negative values) across a dose response for test chemicals. Potencies were determined using EC_20_/EC_50_ values (concentrations of each chemical that exhibit 20%/50% of their own maximal activity) as determined using GraphPad Prism 9.0. Three biological replicates (separate cell passages/assays) were performed for each cell line and test chemical, with four technical replicates at each concentration within each assay.

### 2.4. Zebrafish Housing and Care

Wildtype AB zebrafish (*Danio rerio*) were housed and cared for according to standard protocols and best ethical practices as approved by the North Carolina State University (protocol #IACUC-19-726; approved on 25 October 2019) and the Wayne State University Institutional Animal Care and Use Committees (protocol #IACUC-20-06-2408; approved on 24 August 2020). To generate embryos, ABxAB adult zebrafish were paired in breeding chambers and embryos were collected at the conclusion of the spawning event. Embryos were cleaned and stored overnight in embryo rearing media (ERM) with methylene blue. Zebrafish were fed beginning 6 dpf with GEMMA Micro 75 (Skretting) twice per day until 15 dpf when they were switched to GEMMA Micro 150 until 30 dpf. Fish were maintained in crystallizing dishes in 10–15 mL of ERM until sacrifice, with media changes at least every other day.

### 2.5. Zebrafish Exposures 

At approximately 24 h (1 dpf) following spawns, viable embryos were separated out into 50 mL glass crystallizing dishes in 10 mL of ERM for chemical exposures (*n* = 10–15 individual embryos per chemical test concentration). Chemical exposures were performed in 10 mL of ERM using individual chemical stocks at 0.1% DMSO vehicle. Zebrafish were exposed from 1 dpf through 6 dpf, with complete media and test chemical changes made every 24 h to ensure consistent dosing. Due to the lack of available authentic standards for specific ethoxylate chain lengths, concentrations were not determined in the dosing medium; as such, they should be considered as nominal concentrations only. Following dosing, exposure media was replaced with fresh ERM without test chemicals and fish were aged out to 30 dpf when they were measured and sacrificed.

### 2.6. Zebrafish Metabolic Health Determinations 

The alamar blue assay was performed at 6 dpf according to amended previously published protocols [61,62] to measure zebrafish metabolic rate. This timepoint was selected to address potential chemical-mediated effects on energy expenditure immediately following the cessation of exposures. Briefly, following chemical exposures, zebrafish were transferred to fresh ERM with no added chemicals. For alamar blue testing, two or three wells of *n* = 3 zebrafish each were set up in 24-well black clear-bottom microtiter plates for each test chemical, control, and concentration (*n* = 6–9 fish per exposure group, with two separate exposure experiments). ERM was removed from wells and replaced with 1 mL of alamar blue dye solution (99% embryo media, 1% alamarBlue™ Cell Viability Reagent). Plates were immediately read using an iD5 Molecular Devices plate reader using 530/590 excitation/emission wavelengths. After reading, plates were placed into a 28 °C incubator, protected from light. After one hour, plates were removed from incubator, and fluorescence was measured again using the same wavelengths. Change in fluorescence units was calculated by subtracting background fluorescence for empty wells (dye solution only) and then taking the difference of the values at hour one from those at the start of the exposure. Data are presented as relative change in arbitrary fluorescence units (compared to DMSO-control animals) and comparisons made to the control treatments as described in the statistics subsection.

Following alamar blue readings, fish were returned to group-housing in crystallizing dishes (≤15 fish per dish) and maintained until 30 dpf. At 30 dpf, fish were stained with Nile Red at a concentration of 0.5 μg/mL in embryo media for 30 min, protected from light. Fish were subsequently anesthetized with tricaine, mounted on slides, and imaged using a Leica Thunder M205FA stereoscope. Fish were imaged using a yellow fluorescent protein (YFP) filter at 2× magnification for full body image and standard-length measurements, then imaged at 16× magnification for higher-resolution adipocyte fluorescence quantifications. Following imaging, fish were dry weighed on a microbalance and then snap frozen whole in liquid nitrogen. For adipose quantification, files were exported and imported into Fiji version 2.1.0. A macro was run to isolate the pixels present in the green channel, set thresholding at a consistent level across all study samples, and then quantify the area stained by the Nile Red. Total fish fluorescence was calculated for every fish in each treatment group and concentration. Following quantification, presence of adipose in each of the 34 defined zebrafish adipose depots (described previously [40,41,42]) was scored as presence/absence and compared to DMSO control animals to determine potential dysregulation of specific adipose depot development (Figure S1C). Briefly, previous research has carefully detailed the developmental timeline for each of these 34 adipose depots as well as the histologic, morphologic, and phenotypic features of each depot and their similarities to humans [40,41,42,63]. As rigorous characterization of each depot has not yet been performed, our quantitative assessments have focused on total body fluorescence, and preliminary interrogation of depot-specific differences have focused on qualitative differences.

### 2.7. Statistical Analysis

Cell data are presented as means ± SEM from four technical replicates of three independent biological replicates. Zebrafish growth and metabolic data are presented as means ± SEM from 6–10 technical replicates (individual embryos in same exposure group from one spawn) of four independent biological replicates (independent spawning events). Two-way Kruskal–Wallis with Dunn’s multiple comparisons test was performed to determine significant differences across concentrations and relative to DMSO-control fish (*p* < 0.05 considered significant). Statistical comparisons were made using GraphPad Prism 9.0.

## 3. Results

Nonylphenol and its ethoxylates were assessed for adipogenic activity utilizing two in vitro hMSC models and for obesogenic activity in vivo by utilizing developmental exposures and growth measurements in zebrafish. hMSCs were differentiated for 14 (Zenbio) or 21 (Lonza) days, exposed throughout the provider recommended differentiation period for each cell line, and then stained to measure DNA content, relative to differentiated vehicle control (as markers for proliferative and/or cytotoxic responses), and triglyceride accumulation (relative to maximal rosiglitazone response; total per well and per cell, normalized to DNA content).

### 3.1. Adipogenic Activity of NPEOs in MSC Models

**Figure 1:**
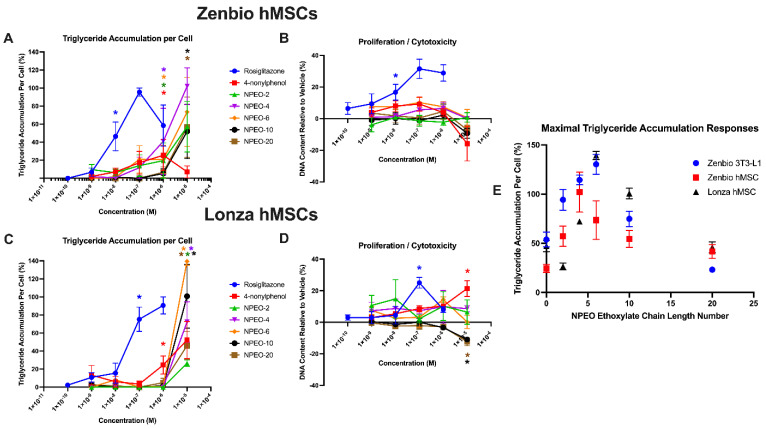
Nonylphenol and polyethoxylates promote adipogenesis in human mesenchymal stem cell models. Zenbio and Lonza human bone marrow–derived mesenchymal stem cell models were differentiated as described in Methods and assessed for adipocyte differentiation (Nile Red staining of lipid accumulation) and cell proliferation (Hoechst staining) after 14/21 (respectively) days of differentiation while exposed to controls chemicals as well as nonylphenol and its ethoxylates. Percent normalized triglyceride accumulation per cell relative to maximal rosiglitazone response (normalized to DNA content) (**A**,**C**). increase (cell proliferation) or decrease (potential cytotoxicity) in DNA content relative to vehicle control (**B**,**D**). Zenbio–sourced cell data provided in (**A**,**B**), and Lonza–sourced cell data provided in (**C**,**D**). Data presented as mean ± SEM from three independent experiments. * indicates lowest concentration with significant increase in triglyceride over vehicle control or cell proliferation/cytotoxicity relative to vehicle control, *p* < 0.05, as per Kruskal–Wallis in GraphPad Prism 9. X–axis format is provided in log scale. Panel (**E**) provides a summary plot of maximal effects on triglyceride accumulation based on ethoxylate chain length across cell models, comparing results from panels (**A**,**C**) with previously published effects in 3T3–L1 cells (PMID: 29106673). NPEO = nonylphenol polyethoxylated (with varying average ethoxylate chain lengths).

Nonylphenol and each of the ethoxylates induced adipogenic effects in both Zenbio and Lonza-sourced hMSCs (Figure 1). In Zenbio-sourced cells, nonylphenol induced 26% triglyceride accumulation relative to the maximal rosiglitazone-induced response at 1 μM (Figure 1A). Ethoxylated nonylphenols promoted increasing activity with increasing ethoxylate chain length up to NPEO-4 (102% triglyceride accumulation at 10 μM) and then decreasing effects down to NPEO-20 (57% triglyceride accumulation at 10 μM). Neither nonylphenol nor any of the ethoxylates significantly induced either pre-adipocyte proliferation or cytotoxicity in Zenbio MSCs (Figure 1B). In Lonza-sourced cells, nonylphenol induced 52% triglyceride accumulation at 10 μM, approximately double the maximum induction in the Zenbio MSCs (Figure 1C). Ethoxylated nonylphenols promoted increasing activity with increasing ethoxylate chain length, with a maximum response for NPEO-6 (139% triglyceride accumulation at 10 μM) and then decreasing effects down to NPEO-20 (46% triglyceride accumulation at 10 μM). Similar pattern of response was observed for triglyceride accumulation when considering responses relative only to the differentiated solvent controls (Figure S2). Nonylphenol promoted significant pre-adipocyte proliferation (21% increased DNA content relative to the differentiated solvent control at 1 μM) and NPEO-10 and 20 induced significant cytotoxicity at 10 μM (Figure 1D). Maximal responses for each test chemical were further compared across cell lines (maximal response concentration varied across each test chemical and cell line, generally 1–10 μM), including both hMSCs examined here, and also 3T3-L1 cells examined previously [36] (Figure 1E). Responses in each cell line exhibited increasing triglyceride accumulation with increasing nonylphenol ethoxylation up to a medium ethoxylate chain length and then decreasing activity up to longer ethoxylate chain lengths. Maximal responses were observed for NPEO-4 in the Zenbio hMSCs and NPEO-6 in the Lonza hMSCs and also the 3T3-L1 cells (Figure 1, Table S1).

### 3.2. Lethality of NPEOs on Zebrafish 

**Figure 2:**
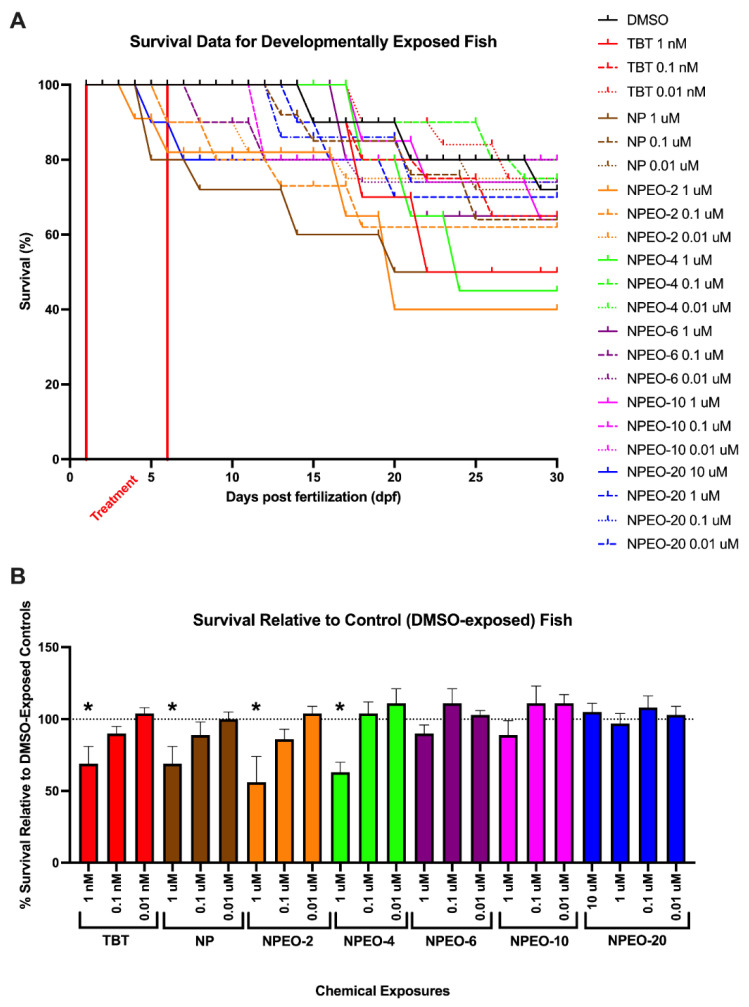
Lethality of nonylphenol and polyethoxylates in developmentally exposed zebrafish. Zebrafish were developmentally exposed to control chemicals, nonylphenol, or nonylphenol polyethoxylates from one through six days post-fertilization. Following exposures, fish were aged to 30 days post-fertilization. Lethality was measured twice daily to determine survivorship across test chemicals and treatments for each test chemical (**A**). *n* = 15 replicate fish in each biological replicate (spawning event) for each test chemical and concentration, and four spawns were performed (four biological replicates) and averaged for responses depicted here, for approximately 60 fish evaluated per experimental group. Lethality is depicted in survivorship curves, with decreasing response on the Y axis depicting greater lethality of the test chemicals. Survival percent relative to DMSO vehicle control exposed fish at 30 days (**B**). * indicates significant change in survival compared to vehicle control fish, *p* < 0.05, as per Kruskal–Wallis test with Dunn’s multiple comparisons. TBT 10 nM, NP 10 μM, NPEO-2 10 μM, NPEO-4 10 μM, NPEO-6 10 μM, and NPEO-10 10 μM promoted absolute toxicity (no surviving fish) and thus are not depicted here for the purposes of clarity. DMSO = dimethylsulfoxide, vehicle control; TBT = tributyltin chloride; NPEO = nonylphenol polyethoxylated (with varying average ethoxylate chain lengths).

Zebrafish were checked twice daily for gross lethality of the test chemicals, to determine non-toxic levels for subsequent analyses. Vehicle (DMSO)-treated fish had average survival of approximately 75% throughout the 30 days, with no mortality observed during the chemical exposure window and limited mortality in the weeks following (Figure 2). At 10 μM, nonylphenol and most NPEOs exhibited complete lethality, except for NPEO-20, where 80% of the exposed fish survived at 30 dpf. The lower-chain length ethoxylates exhibited the greatest toxicity, with survival of only 40–50% of the fish in the 1 μM nonylphenol, NPEO-2, and NPEO-4 groups. Higher ethoxylate chain compounds and lower concentrations of each had lower toxicity, with equivalent survival to the DMSO fish of 65–80%. The TBT positive control was significantly more toxic than NPEOs, with complete lethality noted for concentrations of 10 nM and above, and survival of 50% at the 1 nM concentration. Only concentrations where >50% of fish survived to 30 days were examined for metabolic health endpoints.

### 3.3. Metabolic Health, Growth Trajectory, Weights, and Adipose Deposition 

**Figure 3:**
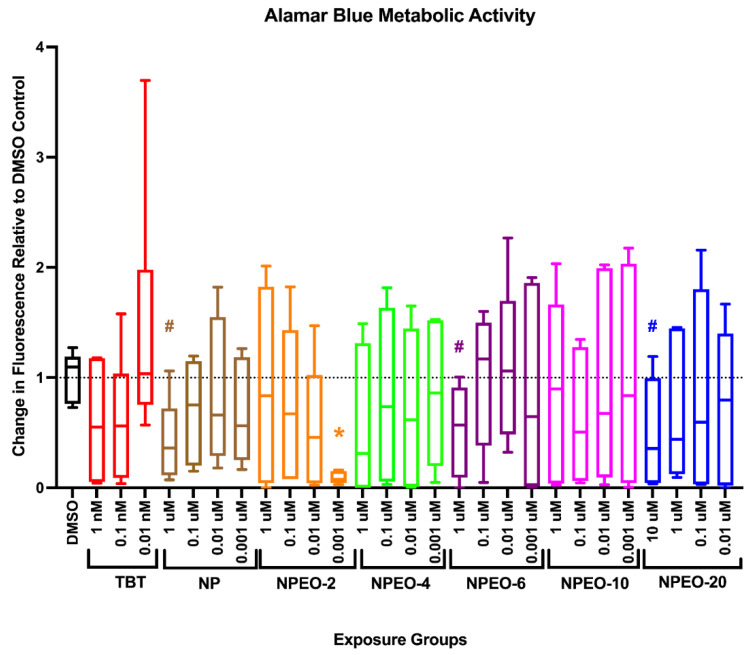
Metabolic activity in zebrafish developmentally exposed to nonylphenol and polyethoxylates. Zebrafish were developmentally exposed to control chemicals, nonylphenol, or nonylphenol polyethoxylates. Immediately following exposure, at six days post-fertilization, metabolic activity was measured using the alamar blue assay. Two groups of three replicate fish were transferred into wells of a 24-well black clear-bottom plate, media removed, and alamar blue solution added to wells. Plates were immediately read for fluorescence, then incubated in the dark for several hours, before measuring fluorescence again. The increase in fluorescence is correlated with increased metabolic activity in the fish. Chemical exposure treated fish responses were compared with dimethylsulfoxide (0.1%, vehicle control) treated fish to determine significant differences. *n* = 6–9 replicate fish in each biological replicate (spawning event), and three spawns were performed for approximately 24 fish per exposure group. * indicates significant change in arbitrary fluorescence compared to vehicle control fish, *p* < 0.05, as per Kruskal–Wallis test with Dunn’s multiple comparisons. DMSO = dimethylsulfoxide, vehicle control; TBT = tributyltin chloride; NPEO = nonylphenol polyethoxylated (with varying average ethoxylate chain lengths). # represents *p* < 0.10, as per statistics described above. Box and whisker plots depict the following metrics: whiskers represent 10–90th percentiles, box bounds represent the 25th to 75th percentiles, and the middle line represents the median.

Energy expenditure of exposed zebrafish was determined through measurement of cumulative NADH_2_ production at 6 dpf via the alamar blue assay as an approximate measure of zebrafish oxidative metabolism [61,62]. The lowest dose (1 nM) of the NPEO-2 had significantly lower metabolic activity than the DMSO-exposed control fish (*p* < 0.05, Figure 3), though no other significant differences were observed. The highest dose (1 μM) for nonylphenol and NPEO-6, as well as 10 μM NPEO-20 tended to have decreased metabolic activity, though these differences were not significant (*p* < 0.10).

**Figure 4:**
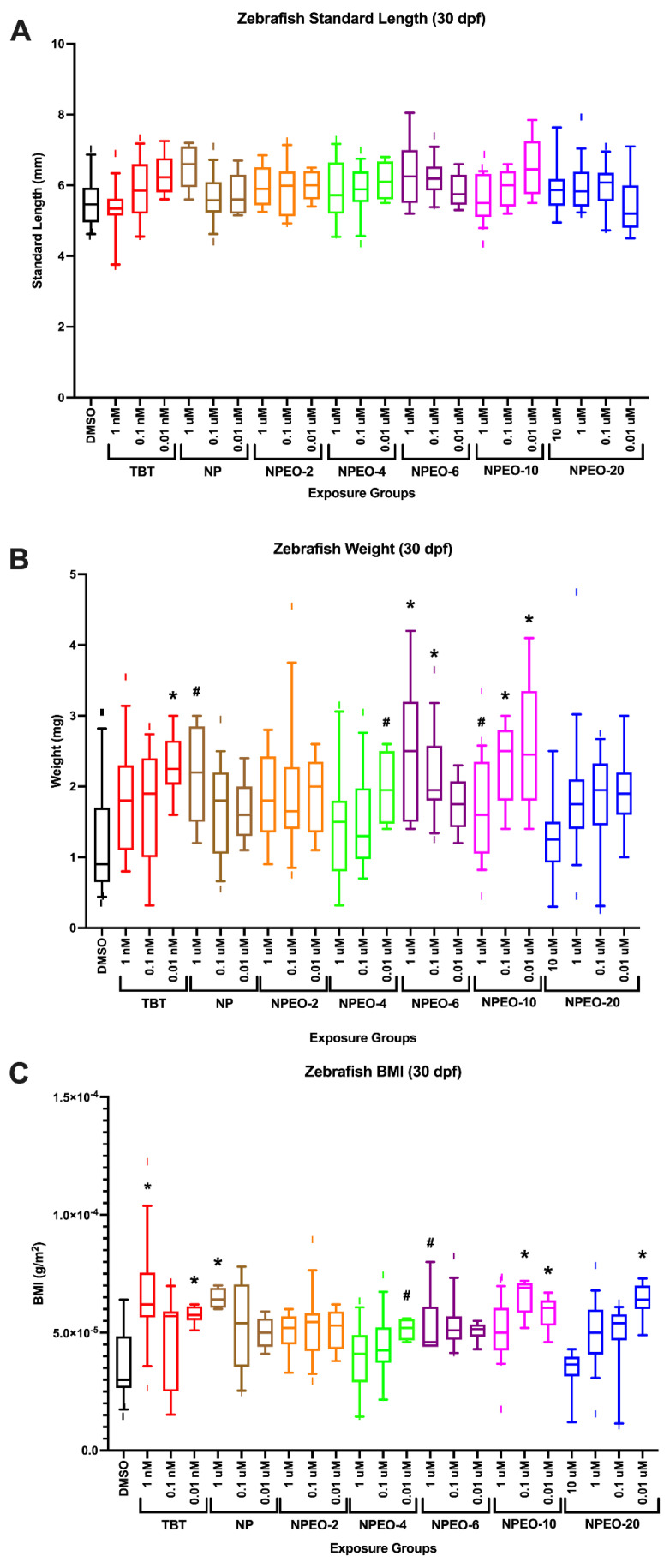
Growth trajectories in zebrafish developmentally exposed to nonylphenol and polyethoxylates. Zebrafish were developmentally exposed to control chemicals, nonylphenol, or nonylphenol polyethoxylates; aged to 30 days post−fertilization; then measured and stained with Nile Red (0.5 μg/mL) for 30 min. Zebrafish were imaged and standard length of each fish was measured (**A**) using the integrated point−to−point measurement tool within the Leica software, which scales by magnification. Following imaging, zebrafish were blotted with kim wipes and weighed on a microbalance to obtain total body weights (**B**) for each fish and then averaged across test chemicals and concentrations. Zebrafish body mass index (**C**) was calculated by dividing the calculated standard length and weights and correcting measurement units to g/m^2^. *n* = 24 (DMSO), 16, 21, 25, 15, 20, 24, 14, 21, 25, 15, 25, 27, 22, 28, 25, 22, 27, 26, 25, 23, 26, and 25 across four spawning events (biological replicates) for exposure groups listed below, respectively. * indicates significant increase in total body fluorescence quantification over vehicle control fish, *p* < 0.05, as per Kruskal–Wallis test with Dunn’s multiple comparisons. DMSO = dimethylsulfoxide, vehicle control; TBT = tributyltin chloride; NPEO = nonylphenol polyethoxylated (with varying average ethoxylate chain lengths). # represents *p* < 0.10, as per statistics described above. Box and whisker plots depict the following metrics: whiskers represent 10–90th percentiles, box bounds represent the 25th to 75th percentiles, and the middle line represents the median.

Developmentally exposed zebrafish (from groups with low/no apparent toxicity) were subsequently aged to 30 dpf, stained with Nile Red fluorescent stain, and then measured, imaged, and weighed. Zebrafish did not exhibit any significant changes in standard length across treatment groups (Figure 4A), suggesting that the size of the fish were not demonstrably different at this early life stage. Despite this, increases in total body weight were observed in several treatment groups (Figure 4B). Tributyltin chloride-exposed fish had increased body weights relative to DMSO control fish (−0.9 mg) at each concentration (1.8–2.3 mg), though was only significantly different in the 0.01 nM exposure group. The high dose of nonylphenol (1 μM) tended to have increased body weights (*p* < 0.10) relative to control fish, as well as the low dose of NPEO-4 (*p* < 0.10). The top two doses of NPEO-6 had significantly increased body weights to controls (*p* < 0.05), and all doses of NPEO-10 tended to be increased (significant at both 0.1 and 0.01 μM). No concentrations of NPEO-2 or 20 induced any significant change in total body mass. A similar pattern was observed when correcting body mass by standard length to create a body mass index measurement (Figure 4C).

**Figure 5:**
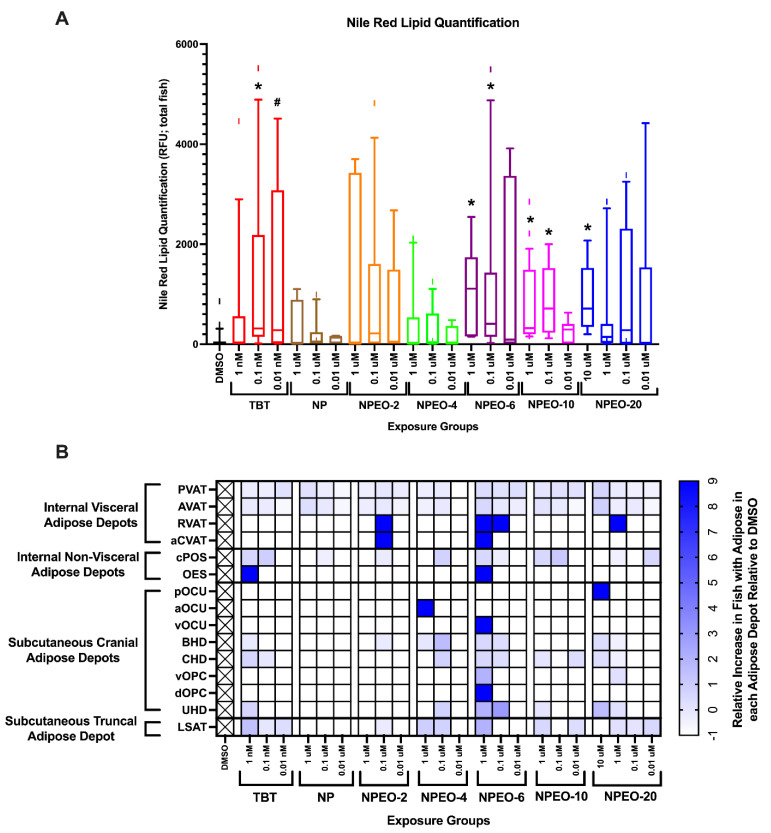
Adipose Deposition in Zebrafish Developmentally Exposed to Nonylphenol and Polyethoxylates. Zebrafish were developmentally exposed to control chemicals, nonylphenol, or nonylphenol polyethoxylates; aged to 30 days post-fertilization; then measured and stained with Nile Red (0.5 μg/mL) for 30 min. Total body fluorescence (**A**) was imaged at 16× magnification using a yellow fluorescent protein filter (representative images in Figure 6) and fluorescence was quantified for each fish and then biological replicates were averaged. *n* = 24 (DMSO), 16, 21, 25, 15, 20, 24, 14, 21, 25, 15, 25, 27, 22, 28, 25, 22, 27, 26, 25, 23, 26, and 25 across four spawning events (biological replicates) for exposure groups listed below, respectively. * indicates significant increase in total body fluorescence quantification over vehicle control fish, *p* < 0.05, as per Kruskal–Wallis test with Dunn’s multiple comparisons. # represents *p* < 0.10, as per statistics described above. Box and whisker plots depict the following metrics: whiskers represent 10–90th percentiles, box bounds represent the 25th to 75th percentiles, and the middle line represents the median. Developmental trajectory of adipose depots across the zebrafish (**B**). Relative proportions of fish exhibiting fluorescing adipocytes in each depot in the DMSO fish were set as “normal” and the heat map depicts increased proportions of fish in each group with visible adipocytes in each depot. Adipose depots labeled as per the developmental guides provided in Minchin and Rawls, 2017 (PMID: 28348140) and grouped based on anatomical classifications. A value of 9 represents a 9X increase in the proportion of fish in an exposure group PVAT = pancreatic visceral adipose tissue; AVAT = abdominal visceral adipose tissue; RVAT = renal visceral adipose tissue; aCVAT = anterior cardiac visceral adipose tissue; cPOS = central paraosseal non-visceral adipose tissue; OES = esophageal non-visceral adipose tissue; LSAT = lateral truncal adipose tissue; pOCU = posterior ocular adipose tissue; aOCU = anterior ocular adipose tissue; vOCU = ventral ocular adipose tissue; BHD = basihyal hyoid adipose tissue; CHD = ceratohyal hyoid adipose tissue; vOPC = ventral opercular adipose tissue; dOPC = dorsal opercular adipose tissue; and UHD = urihyal hyoid adipose tissue. DMSO = dimethylsulfoxide, vehicle control; TBT = tributyltin chloride; NPEO = nonylphenol polyethoxylated (with varying average ethoxylate chain lengths).

**Figure 6:**
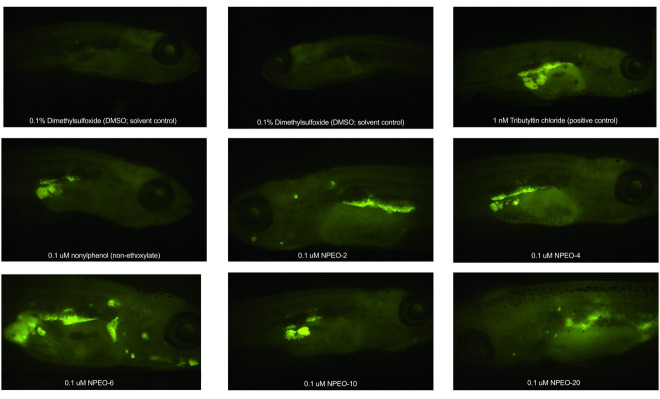
Adipose Patterning in Zebrafish Developmentally Exposed to Nonylphenol and Polyethoxylates. Representative fluorescent images of developmentally exposed zebrafish exposed to control chemicals, nonylphenol, and the nonylphenol polyethoxylates. Anesthetized fish imaged at 30 days post-fertilization, following a 30 min stain (0.5 μg/mL Nile Red). Images obtained at 16× magnification using a yellow fluorescent protein filter. DMSO = dimethylsulfoxide, vehicle control; TBT = tributyltin chloride; NPEO = nonylphenol polyethoxylated (with varying average ethoxylate chain lengths).

Total fluorescence quantification was then performed on all fish to determine total body lipid accumulation across test chemicals and concentrations (Figure 5A and Figure 6). DMSO-control fish had few apparent adipocytes by this age, primarily focused in the pancreatic and abdominal visceral adipose depots (−50–60% of DMSO fish had visible adipocytes in these depots), though certain replicate DMSO-fish had visible adipocytes in the internal non-visceral, subcutaneous truncal, and subcutaneous cranial adipose regions (−10% of DMSO-exposed fish). TBT fish exhibited greater lipid staining than DMSO fish at 0.1 and 0.01 nM (*p* < 0.05 and *p* < 0.10, respectively). NP, NPEO-2, and NPEO-4 groups were not significantly different than the vehicle; 1 and 0.1 μM NPEO-6 and NPEO-10 fish had significantly greater lipid staining relative to control fish; as well as the 10 μM concentration of NPEO-20. Scoring the proportion of each exposure group for the percentage of fish with apparent adipocytes in each of the 34 regionally distinct depots [40,42] (Figure S1C), there was an increased proportion of fish with adipocytes in the pancreatic and abdominal visceral adipose tissues across all test chemicals and treatment groups relative to the DMSO control group (Figure 5B). Particularly exacerbated groups included the renal visceral adipose depot, anterior cardiac visceral adipose tissue, and esophageal non-visceral adipose tissue. NPEO-6 fish in the 1 μM exposure group exhibited the most diverse assortment of adipose tissues, with at least some fish within the group having stained lipid droplets in 13 distinct adipose depots, both subcutaneous and visceral. Preliminary grouping of these depots based on anatomical classification suggests that internal visceral depots may be more impacted than other groupings (e.g., PVAT, AVAT, RVAT, aCVAT). These depots were increased in nearly every exposure group relative to control fish (PVAT, AVAT) or strongly increased in select exposure groups (RVAT, aCVAT). Other particularly impacted depots included LSAT (increased in 12/22 exposure groups), CHD (9/22), and BHD (8/22).

## 4. Discussion

Previous research in our group, using the 3T3-L1 murine pre-adipocyte model, had detailed adipogenic activity for nonylphenol and diverse polyethoxylates [36]. 3T3-L1 cells are routinely used to assess putative adipogenic activity [52,53,64,65]. However, we recently demonstrated poor inter-laboratory reproducibility in chemical responses for the 3T3-L1 cell model [37]. Given these results and the poor maintenance of this cell line over the last several decades (cell providers report very different abilities to differentiate their stock of these cells) [66,67,68], it is important to validate robust and translational models that have more direct relevance to human health outcomes. To that end, herein we evaluated two separate hMSC models and compared the results to previous 3T3-L1 testing [36]. Interestingly, similar response patterns were observed across all three cell lines for triglyceride accumulation (Figure 1E). The maximal response was observed for NPEO-6 in both Zenbio 3T3-L1 and the Lonza hMSCs, and for NPEO-4 in the Zenbio hMSCs. 

While consistent patterns were observed in the triglyceride accumulation response (marker of adipocyte differentiation), consistent responses were not observed in the pre-adipocyte proliferation measure. In 3T3-L1 cells [36], all NPEOs other than NPEO-20 induced significant proliferation (NPEO-4 > NPEO-6 > nonylphenol = NPEO-2 = NPEO-10). In hMSCS, much lower magnitude responses were observed in both cell lines. Nonylphenol promoted a low-level of proliferation in the Lonza hMSCs, though NPEO-10 and 20 promoted significant cytotoxicity, with no other apparent effects on proliferation. The Zenbio hMSCs, in contrast, had no significant effects on either proliferation or cytotoxicity at any of the tested concentrations. We previously reported marked differences in the proliferation measure based on both cell models and cell source used and the brand of tissue culture plate [37,59], suggesting that this may be a more variable measure than triglyceride accumulation. Of note, the 3T3-L1 cells and both hMSC models were differentiated using distinct media formulations specific to hMSC cell line providers (as noted in methods), timelines (Zenbio cells = 14 days; Lonza cells = 21 days; manufacturer-recommended), and differentiation procedures (Zenbio = 7 days differentiation, 7 days maintenance; Lonza = 3 days differentiation, 3 days maintenance, repeated three times) which may have contributed to some of these disparate effects.

We also report for the first time that nonylphenol and its ethoxylates promote weight gain and increased adiposity in the zebrafish model, following developmental exposures. We exposed zebrafish beginning as embryos and through early larval development (1–6 dpf, before the appearance of adipocytes) to various concentrations of the NPEOs in embryo media. We observed increased body weight and adipocyte staining without any significant impacts on standard length. Notably, we observed increasing adipogenic and obesogenic activity with increasing ethoxylate chain lengths up to maximums at NPEO-6 and then decreasing activity with the longest ethoxylate chain lengths.

Limited prior research has evaluated these chemicals for effects on growth and weight. Previous research exposed rainbow trout from hatching for 35 days and then followed through 466 days. While decreased weights were observed at 108 days for exposed fish, increased body weights were observed for 10 μg/L nonylphenol at 466 days and for both 1 and 10 μg/L nonylphenol mono-carboxylic acid at 220, 300, and 466 days [33]. No apparent effects were observed for NPEO-2, and no apparent modulation of body length were observed. In addition to the separate fish species, the concentrations used in this previous research (1–30 μg/L, 0.005–0.14 μM) were generally lower than examined in the present study. These researchers suggested that these effects on body weight may be occurring through activation of the estrogen receptor, which has been well-described for both nonylphenol and the NPEOs [28,69], but the underlying mechanisms of these effects have not yet been rigorously examined. While not putatively significant, a trend for increased body weights was observed following 100 days of exposure to nonylphenol and polyethoxylates in Japanese medaka [28]. Importantly, we have not definitely determined the causal mechanisms for the effects reported herein, though exposure to estrogenic chemicals has certainly been well described to promote growth in other species [70,71,72]. Ongoing research efforts should carefully examine pro-adipogenic mechanisms through controlled testing in vitro and genetic manipulations in vivo to conclusively identify causal/contributory pathways.

The utility of the zebrafish model in evaluating potential obesogens has been described previously [39]. This model was proposed to utilize a high fat diet and chemical exposures over a short duration in developed zebrafish to evaluate potential obesogens, though more recent research has utilized earlier developmental exposures. Herein, we utilized a developmental exposure model, with our chemical exposures spanning embryonic and early larval development, with subsequent analysis of health outcomes several weeks later. Moreover, while many other publications have assessed Nile Red staining in very early life (≤15 dpf) [50,73,74], this is (in some cases) before or very shortly after adipocytes are reported to develop under normal conditions [40,41,42]. Herein we evaluated adipose staining at 30 dpf, similar to several other publications [46,48,51], which allows for not only visualization/quantification of larger adipose depots but also characterization and analysis of the presence of adipose within specific depots. This will allow for future research to potentially tease apart differential susceptibility by adipose depot, which is yet undetermined.

Preliminary analyses suggested that internal visceral depots may be more impacted than other groupings, though this requires further substantiation. PVAT and AVAT, the most impacted depots, are also the first depots to develop in the zebrafish [40,41,42]. Notably, other widely impacted depots (BHD, CHD, RVAT) are also earlier developing depots, though importantly have often developed before the standard developmental length. In contrast, other depots such as UHD or dOPC have developed in some exposed fish long before they should normally have developed based on standard length (as measure of developmental timepoint). Visceral adiposity has a greater connection with metabolic disruption and disease pathology, as reviewed previously [75], supporting a need for future assessments that consider specific depots and classifications of adipose. Previous research has described highly conserved morphology, energy storage and lipid depot development, and associated/underlying gene signaling across vertebrates (e.g., between zebrafish and humans) [40,41,42,63]. Specifically, the same distinguishing histological, morphological, and phenotypic features of human white adipocytes have also been described in zebrafish (e.g., unilocular lipid droplet with displaced nucleus, molecular markers [adipokines, proteins, and gene expression] of differentiated adipocytes, and with morphology of adipose depot divisions and subdivisions) [40,41,42,63].

Interestingly, we report obesogenic effects in our zebrafish model that are consistent with the adipogenic effects observed through the in vitro testing. The three cell models we have evaluated reported the greatest adipogenic effects at NPEO-4 and NPEO-6, with decreasing activity with increasing and decreasing chain length from there. For each of the zebrafish measures (gross weight, BMI, and fluorescence quantification), we also observed the greatest effects in the NPEO-6 exposure group, though effects were also observed in the NPEO-10 and sometimes in the NPEO-20 exposure group. However, it should be noted that appreciable effects were only observed at the 10 μM NPEO-20 group, which is the only 10 μM exposure group that was not overtly toxic to the fish. So, while the three in vitro models achieved consistent effects, they were also predictive of the in vivo outcomes we measured in our developmentally exposed zebrafish. This is promising for risk assessment strategies, given the greater reliance on in vitro and non-mammalian vertebrate in vivo models. Increasing tools, such as the energy expenditure assessments possible through the alamar blue assay, have continued to improve the breadth of metabolic health testing possible using this model.

There are some inherent limitations to the research we present here. Importantly, we still have an incomplete understanding of the underlying mechanisms driving these effects. As we noted previously [36], some previous results have reported decreasing toxicity with increasing ethoxylate chain length [13,14], which has suggested lower environmental toxicity for longer chain ethoxylated surfactants. It’s possible that a short ethoxylate chain allows for greater lipophilicity, whereas the longer tails inhibit membrane permeability, limiting nuclear receptor activation. Moreover, none of the ethoxylates included here have commercially available pure standards; instead, we have utilized commercial mixtures with average ethoxylate chain lengths as specified here. This limits analytical characterization of these in our exposure dishes and limits environmental characterization of relevant concentrations, particularly for the longer chain length ethoxymers. This also makes careful ethoxylate chain length assessments slightly problematic, as we cannot separate specific ethoxymers for toxicological testing. As such, while we report the greatest effects in the average chain length range of 4–6, these compounds are also present in the lower activity NPEO-10 and NPEO-20; this suggests that these longer chain length chemicals may have even less adipogenic and/or obesogenic activity, as a portion of the activity is likely to be contributed by the shorter chain-length ethoxymers that are present at lower proportions in those mixtures. It is also worth noting that inhibited energy expenditure was observed for some chemicals where significant toxicity was also observed; generally, this occurred before significant lethality was observed in the fish but should be examined further as a potential mediating factor in the decreased energy expenditure for certain chemicals.

APEOs such as the NPEOs are still used widely (particularly in consumer products, as regulations and/or voluntary replacements have focused on industrial applications). These compounds are routinely detected in the environment [76]; at μg/L concentrations (−1–10 nM, for the most potent NPEOs) in wastewater and other surface water sources, and at ng/L (−1–10 pM) concentrations in drinking water. They have also been detected in the indoor environment and appear to accumulate in indoor house dust [22], representing an additional source of chronic human exposure. This research suggests metabolic health concerns for these widespread and newly reported chemical obesogens, suggesting further need to assess molecular mechanisms and better characterize environmental concentrations as well as human exposure levels for human health risk assessments. There is also a growing use of the alcohol ethoxylates and other alternative products recommended by the US Environmental Protection Agency [77], as well as new reports of ethoxylated PFAS compounds being used as replacements. Toxicity information for these compounds is much more limited and requires further evaluation in future studies.

## Figures and Tables

**Table 1:** Alkylphenol Ethoxylates and Control Chemicals.

Chemical	Acronym	CAS #	Manufacturer	Catalog #	Lot #	Avg MW	Density	Molecular Formula
**Alkylphenols/ethoxylates**								
4-nonylphenol	NPEO(0)	84852-15-3	Acros Organics	416,240,010	A0216749	220.4	0.94	C_15_H_24_O
nonylphenol ethoxylate (1–2)	NPEO(1–2)	N/A	Chem Service	S-346	270–35A	294	1.01	C_15_H_24_O(C_2_H_5_O)_1–2_
nonylphenol ethoxylate (4)	NPEO(4)	N/A	Chem Service	S-347	348–75A	396	1.02	C_15_H_24_O(C_2_H_5_O)_4_
nonylphenol ethoxylate (6)	NPEO(6)	N/A	Chem Service	S-348	195–130C	484	1.04	C_15_H_24_O(C_2_H_5_O)_6_
nonylphenol ethoxylate (9–10)	NPEO(9–10)	N/A	Chem Service	S-350	267–60C	602.8	1.06	C_15_H_24_O(C_2_H_5_O)_9–10_
nonylphenol ethoxylate (20)	NPEO(20)	N/A	Chem Service	S-354	127–80C	1101	1.13	C_15_H_24_O(C_2_H_5_O)_20_
*Control chemicals*								
Tributyltin chloride	TBT	1461-22-9	Sigma	442,869	-	325.51	N/A	[CH_3_(CH_2_)_3_]_3_SnCl
Dimethylsulfoxide	DMSO	67-68-5	Sigma	34,869–100 mL	-	78.13	1.10	(CH_3_)_2_SO

Chemical identification, ordering information, and basic physicochemical properties for each of the alkylphenols, ethoxylates, and control chemicals examined in this study. Molecular formulae contain base carbon number as well as average ethoxylate chain number. CAS # = Chemical Abstract Service number

## Data Availability

The data presented in this study are available on request from the corresponding author.

## Supplementary Materials

The following supporting information can be downloaded at: https://www.mdpi.com/2305-6304/10/2/99/s1, Table S1: Adipogenic Effects in Human Mesenchymal Stem Cell Models; Figure S1: Adipogenic and Obesogenic Characterization; Figure S2: Fold Induction of Adipogenic Responses in hMSCs.
