# Correction: Kassotis et al. Nonylphenol Polyethoxylates Enhance Adipose Deposition in Developmentally Exposed Zebrafish. *Toxics* 2022, *10*, 99

**DOI:** 10.3390/toxics10070345

**Published:** 2022-06-22

**Authors:** Christopher D. Kassotis, Matthew K. LeFauve, Yu-Ting Tiffany Chiang, Megan M. Knuth, Stacy Schkoda, Seth W. Kullman

**Affiliations:** 1 Institute of Environmental Health Sciences and Department of Pharmacology, Wayne State University, Detroit, MI 48202, USA; mlefauve@wayne.edu (M.K.L.); yutingtc@wayne.edu (Y.-T.T.C.); 2 Lineberger Comprehensive Cancer Center, University of North Carolina School of Medicine at Chapel Hill, Chapel Hill, NC 27514, USA; mmknuth@ad.unc.edu; 3 Department of Genetics, University of North Carolina School of Medicine at Chapel Hill, Chapel Hill, NC 27514, USA; 4 Toxicology Program, North Carolina State University, Raleigh, NC 27695, USA; sschkod@ncsu.edu (S.S.); swkullma@ncsu.edu (S.W.K.)

## Error in Figure

In the original publication [1], there was a mistake in Figure 6 as published. In creating a representative image of adipose depots from various chemical-exposed fish, one of these images was inadvertently duplicated. This in no way impacted the results or quantitative data, as this figure was intended as a visual depiction of representative data only. The corrected Figure 6 appears below.

The authors apologize for any inconvenience caused and state that the scientific conclusions are unaffected. This correction was approved by the Academic Editor. The original publication has also been updated.

## Figures and Tables

**Figure 6:**
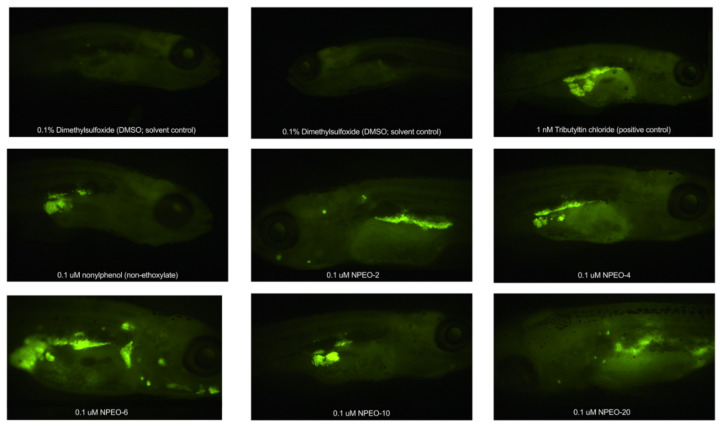
Adipose Patterning in Zebrafish Developmentally Exposed to Nonylphenol and Polyethoxylates. Representative fluorescent images of developmentally exposed zebrafish exposed to control chemicals, nonylphenol, and the nonylphenol polyethoxylates. Anesthetized fish imaged at 30 days post-fertilization, following a 30 min stain (0.5 μg/mL Nile Red). Images obtained at 16× magnification using a yellow fluorescent protein filter. DMSO = dimethylsulfoxide, vehicle control; TBT = tributyltin chloride; NPEO = nonylphenol polyethoxylated (with varying average ethoxylate chain lengths).
