# Supplementary material for: Nonylphenol Polyethoxylates Enhance Adipose Deposition in Developmentally Exposed Zebrafish
Source: Toxics. 2022 Feb 20;10(2):99. doi: 10.3390/toxics10020099 (PMC8879477; doi:10.3390/toxics10020099)
Supplement: Supplementary file 1 [file toxics-10-00099-s001.zip › toxics-1537648-SI.pdf]

toxics MDPI

Supplementary Materials: Nonylphenol Polyethoxylates Enhance Adipose Deposition in Developmentally Exposed Zebrafish

Christopher D. Kassotis, Matthew K. LeFauve, Yu-Ting Tiffany Chiang, Megan M. Knuth, Stacy Schkoda and Seth W. Kullman

Table S1. Adipogenic Effects in Human Mesenchymal Stem Cell Models

Chemical	Zenbio hMSCs				Lonza hMSCs			
	LOEL (µM)	% TG Max	EC_{20} (µM)	% Prolif Max	LOEL (µM)	% TG Max	EC_{20} (µM)	% Prolif Max
Nonylphenol	1.0	25.5%	0.2	9.0%	1.0	52.2%	0.6	21.3%
NPEO-2	1.0	57.1%	1.0	0.0%	10	26.5%	6.0	14.8%
NPEO-4	1.0	102%	0.2	6.5%	10	72.2%	1.9	8.7%
NPEO-6	1.0	73.5%	0.1	10%	10	139.3%	1.4	14.3%
NPEO-10	10	51.9%	2.0	0.0%	10	100.7%	1.5	0.0%
NPEO-20	10	56.7%	1.9	3.8%	10	45.9%	2.3	0.0%

Triglyceride accumulation (percent relative to rosiglitazone-induced maximum) and pre-adipocyte proliferation (percent relative to vehicle control) provided as maximum efficacies. Potencies for each adipogenic mechanism provided as EC_{20} values in µM (concentration at which the chemical exhibits 20% of its maximal activity, respectively). LOEL = lowest observed effect level, the lowest test concentration in µM that exhibited significant activity via either triglyceride accumulation (normalized to DNA content) or pre-adipocyte proliferation.

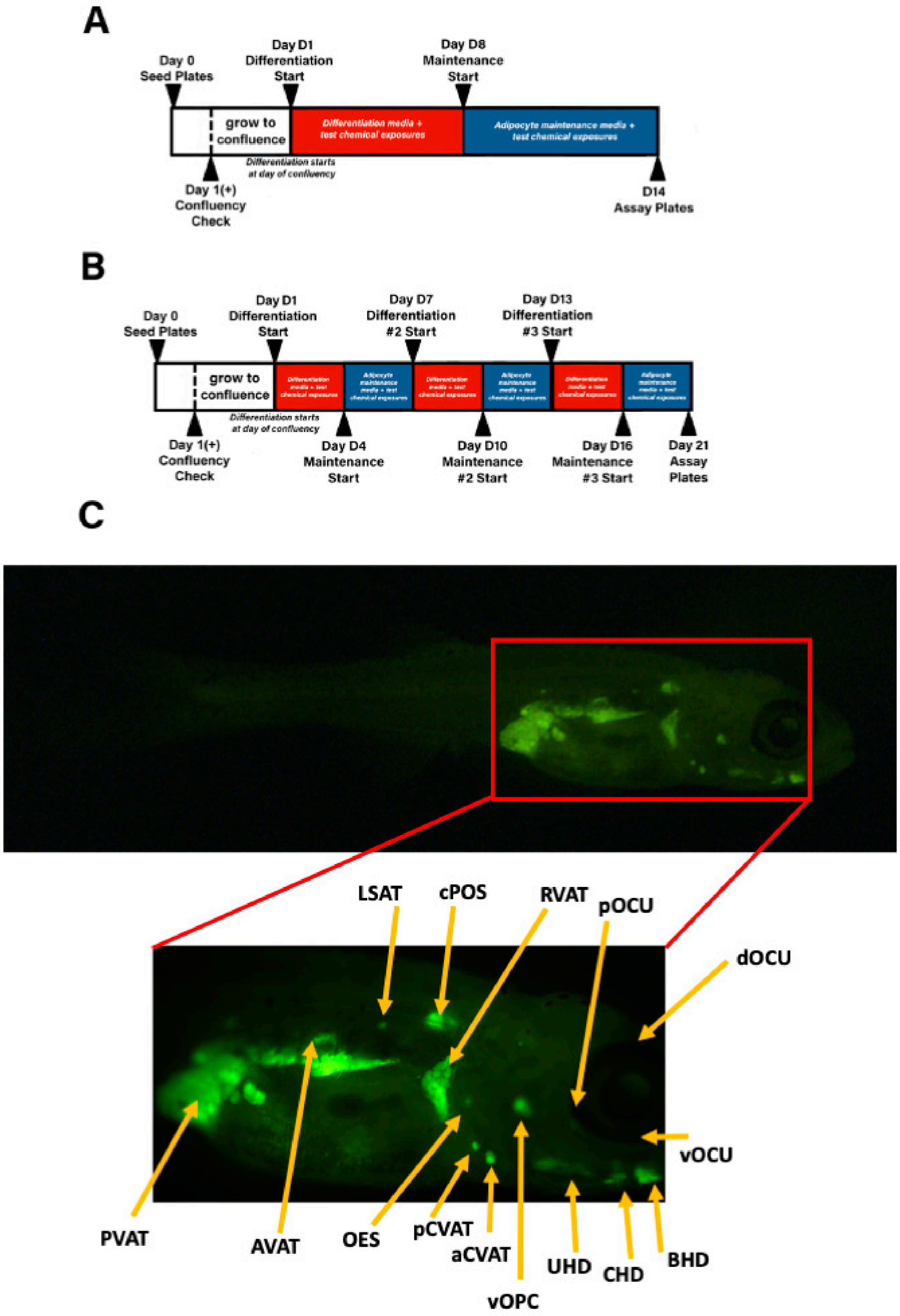

Figure S1. Adipogenic and Obesogenic Characterization . Schematics of the adipogenic differentiation protocol followed for the human bone marrow derived mesenchymal stem cells from Zenbio (**A**) and from Lonza (**B**). Developmental trajectory of adipose depots across the zebrafish (**C**). Approximate locations of depots as defined in results and following the developmental guides provided in Minchin and Rawls, 2017 (PMID: 28348140). Orange lines specify the location of specific adipose depots. Red box describes the overall location within the fish the adipose tissues are located.

PVAT = pancreatic visceral adipose tissue; AVAT = abdominal visceral adipose tissue; RVAT = renal visceral adipose tissue; aCVAT = anterior cardiac visceral adipose tissue; cPOS = central paraosseal non-visceral adipose tissue; OES = oesophageal non-visceral adipose tissue; LSAT = lateral truncal adipose tissue; pOCU = posterior ocular adipose tissue; aOCU = anterior ocular adipose tissue; vOCU = ventral ocular adipose tissue; BHD = basihyal hyoid adipose tissue; CHD = ceratohyal hyoid adipose tissue; vOPC = ventral opercular adipose tissue; dOPC = dorsal opercular adipose tissue; and UHD = urihyal hyoid adipose tissue. DMSO = dimethylsulfoxide, vehicle control; TBT = tributyltin chloride; NPEO = nonylphenol polyethoxylated (with varying average ethoxylate chain lengths).

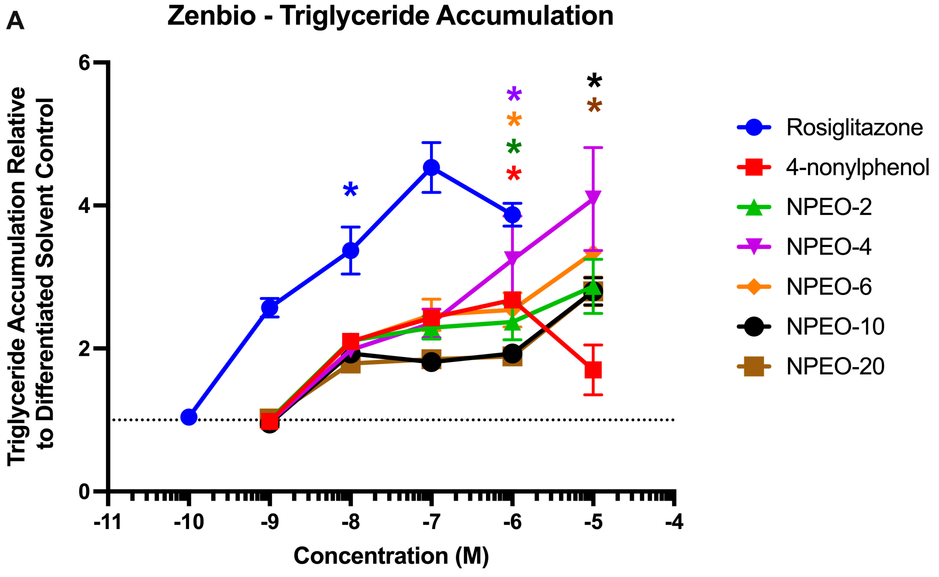

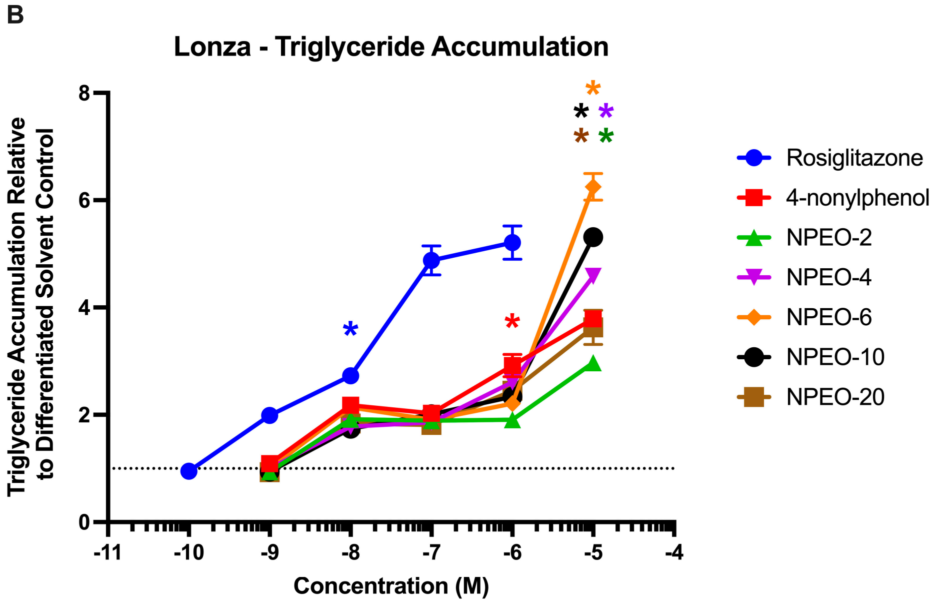

Figure S2. Fold Induction of Adipogenic Responses in hMSCs. Zenbio and Lonza human bone marrow-derived mesenchymal stem cell models were differentiated as described in Methods and assessed for adipocyte differentiation (Nile Red staining of lipid accumulation) after 14/21 (respectively) days of differentiation while exposed to controls chemicals, nonylphenol, and its ethoxylates. Fold induction of responses relative to the differentiated solvent control (0.1% DMSO) cells are provided for Zenbio (**A**) and Lonza (**B**) human mesenchymal stem cells. Data presented as mean ± SEM from three independent experiments. * indicates lowest concentration with significant increase in triglyceride over vehicle control or cell proliferation/cytotoxicity relative to vehicle control, $p<0.05$,

as per Kruskal-Wallis in GraphPad Prism 9. NPEO = nonylphenol polyethoxylated (with varying average ethoxylate chain lengths).
